# Medullary thyroid cancer arising from a thyroid rest: a case report

**DOI:** 10.1093/jscr/rjaf613

**Published:** 2025-08-10

**Authors:** Jacob Beiriger, Hilary C McCrary, Patrick Carpenter, Benjamin L Witt, Devaprabu Abraham, Marcus M Monroe

**Affiliations:** Department of Otolaryngology – Head and Neck Surgery, University of Utah School of Medicine, 30 N. Mario Capecchi Drive, 4th Floor, Salt Lake City, UT 84112, United States; Department of Otolaryngology – Head and Neck Surgery, University of Utah School of Medicine, 30 N. Mario Capecchi Drive, 4th Floor, Salt Lake City, UT 84112, United States; Department of Otolaryngology – Head and Neck Surgery, University of Utah School of Medicine, 30 N. Mario Capecchi Drive, 4th Floor, Salt Lake City, UT 84112, United States; Department of Pathology, University of Utah School of Medicine, 15 North Medical Drive East, Ste. #1100, Salt Lake City, UT 84112, United States; Department of Internal Medicine, Division of Endocrinology, University of Utah School of Medicine, 30 North Mario Capecchi Dr, 3rd Floor North, Salt Lake City, UT 84112, United States; Department of Otolaryngology – Head and Neck Surgery, University of Utah School of Medicine, 30 N. Mario Capecchi Drive, 4th Floor, Salt Lake City, UT 84112, United States

**Keywords:** medullary thyroid carcinoma, thyroid rest, neuroendocrine tumors

## Abstract

Medullary thyroid carcinoma (MTC) is rare and originates from parafollicular C cells and most cases present with a primary thyroid lesion. This report describes a 67-year-old woman with a left-sided neck mass and no evidence of intrathyroidal disease. Positron emission tomography–computed tomography (PET-CT) revealed paratracheal lymphadenopathy; other imaging and TSH were unremarkable. Fine-needle aspiration was inconclusive, and excisional biopsy suggested high-grade metastatic neuroendocrine carcinoma, initially suspected to be pulmonary due to thyroid transcription factor-1 (TTF-1) positivity. Pathology review raised concern for MTC. Endocrine evaluation showed elevated calcitonin (25.4 pg/ml) and carcinoembryonic antigen (CEA) (20.1 ng/ml). She denied personal or family history of thyroid disease or multiple endocrine neoplasia syndromes. Total thyroidectomy with central neck dissection was performed. All thyroid sections stained negative for calcitonin, excluding C cell hyperplasia or intrathyroidal MTC. Lymph node morphology and immunoprofile supported metastatic MTC arising from a thyroid rest. This is a rare entity, with only two other cases documented in the literature.

## Introduction

Medullary thyroid carcinoma (MTC) accounts for approximately 5%–8% of thyroid cancers [1]. It arises from calcitonin-producing parafollicular C cells located between the basal layer and follicular cells [1]. About 25% of MTCs are caused by gain-of-function mutations in the RET proto-oncogene, with C cell hyperplasia often preceding malignancy [2]. Hereditary MTC may occur as part of multiple endocrine neoplasia (MEN) type 2A or 2B syndromes [3]. Sporadic MTC is more common and frequently involves somatic rearranged during transfection (RET) mutations in 40%–60% of cases [2]. Patients typically present in their 50s or 60s with a palpable neck mass [3]. Posterior thyroid lesions may cause compressive symptoms such as hoarseness, dysphagia, or respiratory distress [3]. Fine-needle aspiration (FNA) is commonly used for diagnosis, with pathology demonstrating abundant C cells, fibrovascular bands, salt-and-pepper chromatin, and calcitonin-derived amyloid deposits [4]. Most cases feature a discernible thyroid lesion, but this case illustrates MTC without identifiable disease in the thyroid gland. Only two similar cases are documented. We describe a patient with MTC arising from a thyroid rest and review the diagnostic and management approach.

## Case report

**Figure 1:**
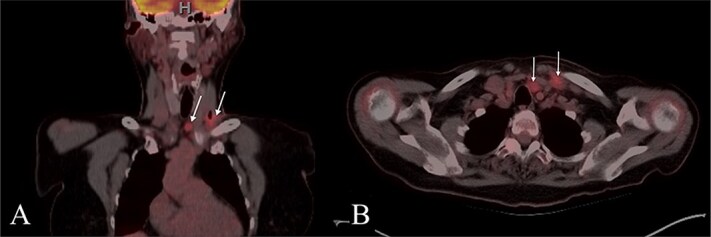
PET CT with coronal (A) and axial (B) images showing enlarged and hypermetabolic level IV and VI lymph nodes with no primary malignancy identified.

A 67-year-old woman with no significant medical history presented to her primary care provider with a growing mass at the left base of the neck. Physical exam revealed an enlarged left supraclavicular node. PET-CT showed paratracheal lymphadenopathy but was otherwise unremarkable (Fig. 1). Chest and abdominal imaging were negative, and thyroid stimulating hormone (TSH) was normal. She was referred to otolaryngology for further evaluation.

FNA was inconclusive. Excisional biopsy demonstrated metastatic high-grade neuroendocrine carcinoma. Histology showed variably sized cell nests with central necrosis, large cells with vesicular nuclei and prominent nucleoli. Immunohistochemistry was positive for CK7, chromogranin, synaptophysin, and TTF-1, initially suggesting a pulmonary origin. Pathology reassessment raised concern for MTC.

**Figure 2:**
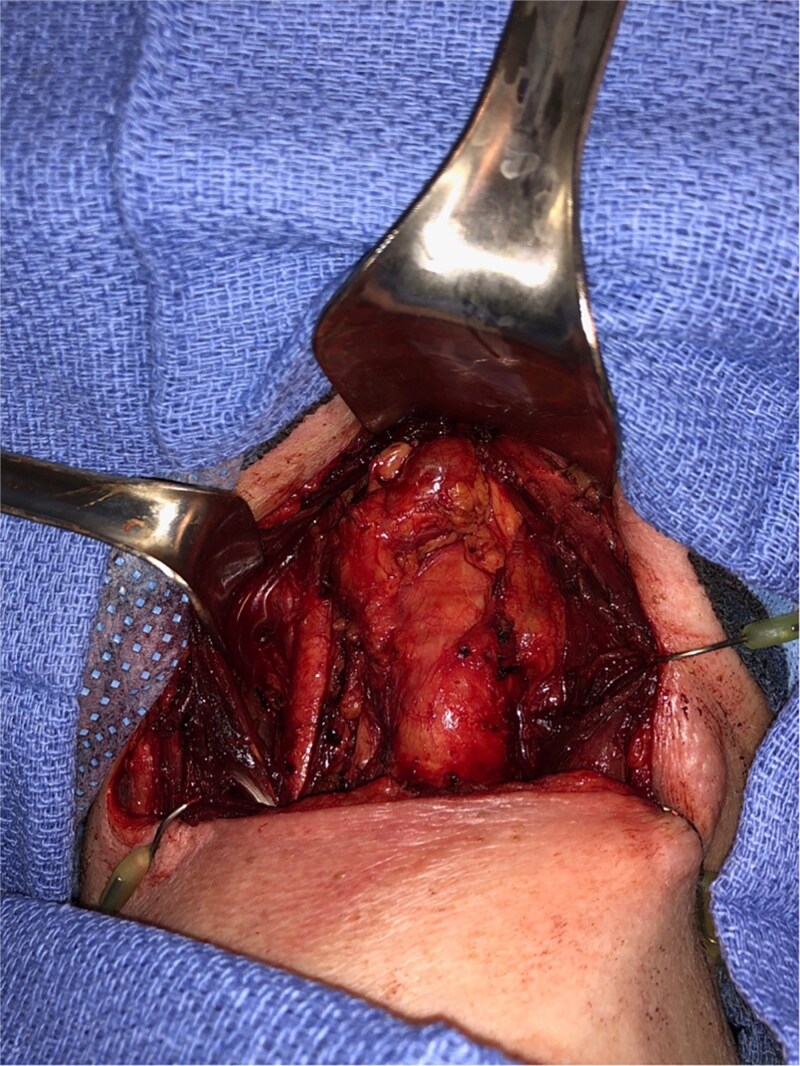
Intraoperative image, demonstrating a central level 6 neck mass medial to the thymus, following total thyroidectomy.

Endocrinology workup revealed calcitonin of 25.4 pg/ml (ref: 0.0–5.1) and CEA of 20.1 ng/ml (ref: 0.0–3.0). The patient denied any family history of thyroid or parathyroid disease or MEN syndromes. She was referred to head and neck surgery and underwent total thyroidectomy with central compartment dissection (Fig. 2).

**Figure 3:**
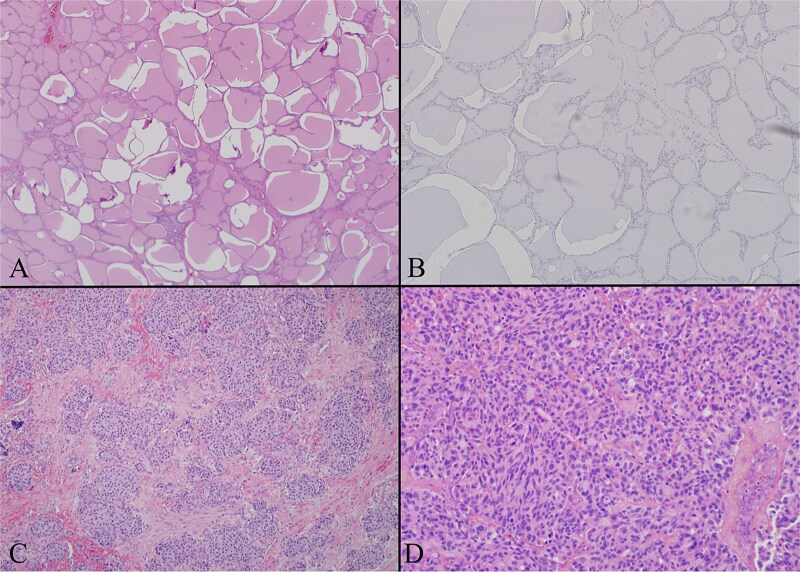
(A) Thyroid gland on low power H + E (4×) photomicrograph showing a representative section of unremarkable thyroid gland. The entire gland had a similar appearance. (B) Thyroid gland calcitonin stain (20×) image showing the thyroid proper is negative for calcitonin. Several calcitonin stains were performed and all were negative excluding C cell hyperplasia and primary medullary carcinoma. (C) Left level 4 lymph node with low power H + E (4×) photomicrograph demonstrating a lateral neck node replaced by tumor. The tumor has a paraganglioma-like appearance with uniform cells in a nested pattern. (D) Left level 6 lymph node with low power H + E (4×) photomicrograph demonstrating a central neck node, replaced by tumor. The tumor has a more classic appearance of medullary thyroid carcinoma here with evident spindle cell morphology.

**Figure 4:**
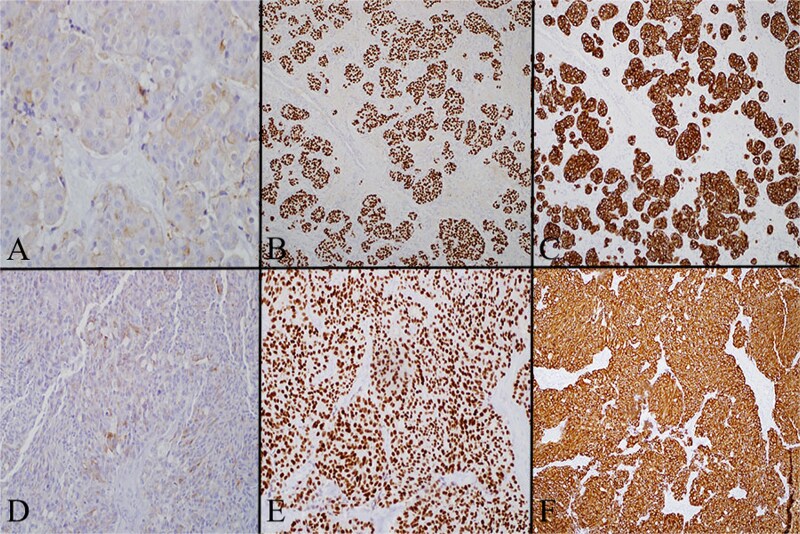
(A) Left level 4 lymph node stained for calcitonin on high power (20×), which demonstrates that in the lateral neck node tumor cells have cytoplasmic expression of calcitonin. (B) Left level 4 lymph node stained for TTF1 on low power (4×) showing that in the lateral neck node the tumor cells are diffusely positive for TTF. (C) Left level 4 lymph node stained for cam 5.2 on low power (4×) showing that in the lateral neck node the tumor cells are diffusely positive for cam 5.2 (pancytokeratin). (D) Level 6 lymph node stained for calcitonin on high power (20×) showing that the central neck node tumor cells have cytoplasmic expression of calcitonin. (E) Level 6 lymph node stained for TTF on low power (4×) showing that the central neck node tumor cells are diffusely positive for TTF. (F) Level 6 lymph node stained for cam 5.2 on low power (4×) showing that the central neck node tumor cells are diffusely positive for cam 5.2 (pancytokeratin).

Multiple thyroid sections were stained for calcitonin to assess for C cell proliferation. All were negative, excluding intrathyroidal MTC or C cell hyperplasia (Fig. 3). Stains of lymph nodes showed patchy but convincing positivity for calcitonin, Cam 5.2, TTF-1, and synaptophysin. S100, GATA3, and Pax-8 were negative (Fig. 4). Morphology revealed nodal deposits with paraganglioma-like and solid spindled patterns, consistent with MTC arising from a thyroid rest. Germline genetic testing was recommended but has not completed.

## Discussion

This case represents only the third documented instance of MTC originating from a thyroid rest. The likely mechanism is failed incorporation of parafollicular C cells into the thyroid during embryologic descent. Although the paratracheal lymph node exhibited PET avidity and histologic features of MTC, it was not considered a primary tumor given its anatomic location outside the thyroid and complete absence of intrathyroidal disease. Rather than an occult thyroidal primary, these findings support the interpretation of metastatic MTC arising from ectopic C cell tissue. Negative calcitonin staining in the thyroid proper supports this hypothesis.

Thyroid rests, also referred to as ectopic thyroid tissue, are remnants of thyroid migration often attached to the lower thyroid pole or found along the path of descent. These remnants can be overlooked during thyroid surgery or misidentified as lymph nodes or parathyroid glands [5]. Importantly, malignancies arising from thyroid rests have been reported, most commonly in the context of papillary thyroid carcinoma [6]. This case extends that paradigm to medullary thyroid carcinoma.

Accurate diagnosis requires distinguishing MTC from other neuroendocrine tumors that secrete calcitonin. These tumors can arise in organs throughout the body and may metastasize to the thyroid or cervical lymph nodes, mimicking primary thyroid malignancies [7, 8]. Immunohistochemistry remains paramount in distinguishing primary versus metastatic neuroendocrine tumors. Metastatic lesions are often negative for both calcitonin and CEA [9]. An exception is small cell lung carcinoma, which may be calcitonin-positive but typically lacks CEA expression. Composite tumors with mixed medullary and follicular features must also be considered; however, these demonstrate thyroglobulin positivity in the follicular component, which was absent in our case [9]. Insular carcinoma and thyroid paraganglioma, also considered, were excluded due to the lack of calcitonin expression [9].

Distinguishing MTC from other metastatic neuroendocrine tumors (NETs) remains a well-documented diagnostic challenge. Particularly, in ectopic or extranodal presentations. Both tumor types can display nested or trabecular architecture and are typically positive for general neuroendocrine markers such as chromogranin and synaptophysin. MTC is generally distinguished by expression of calcitonin and CEA. However, aberrant marker expression or poorly differentiated morphology can obscure diagnosis. Moreover, metastatic NETs to the thyroid region may be negative for calcitonin or only focally express CEA. This further complicates the pathologic distinction. Recent guidelines emphasize the importance of a comprehensive immunohistochemical panel—combining calcitonin, CEA, TTF-1, and site-specific transcription factors—in cases with ambiguous histology. Accurate diagnosis often requires coordination with experienced endocrine pathologists, as histologic heterogeneity in MTC can result in false-negative fine-needle aspiration or misclassification [10, 11].

Notably, this patient had a discordance between CEA and calcitonin levels. While CEA lacks specificity for MTC, it is valuable for monitoring disease progression [12]. A potential hook effect, in which excessively high calcitonin levels yield falsely low immunoassay results, was ruled out through serial dilution testing. Diagnosis was confirmed by an independent laboratory.

Three prior cases highlight the diagnostic and management challenges posed by ectopic MTC. One described a submandibular mass ultimately identified as MTC; thyroid lobectomy revealed only benign adenomatous changes [13]. Another case involved a patient with a lingual thyroid presenting with dysphagia and confirmed MTC [14]. Postoperatively, the patient developed hypothyroidism, and imaging confirmed the absence of orthotopic thyroid tissue. Both cases support the hypothesis that embryonic remnants may harbor parafollicular cells capable of neoplastic transformation.

A separate report detailed a pedunculated upper esophageal mass with cervical nodal metastasis and elevated calcitonin [15]. Though initially concerning for MTC, the tumor was ultimately classified as a carcinoid neuroendocrine tumor. This example highlights the risk of overdiagnosis and suggests that total thyroidectomy is not always warranted without confirmed thyroid disease. However, in our case, surgery was indicated to definitively exclude occult disease and ensure comprehensive staging. Given the absence of a thyroid primary and the relatively low preoperative calcitonin level, bilateral lateral neck dissection was not pursued. Instead, central compartment dissection was performed for staging. Further management was deferred pending postoperative biomarker trends. Calcitonin was undetectable and CEA had normalized and remained stable at the 3-month postoperative follow-up.

## Conclusion

We report a rare case of MTC arising from a thyroid rest, with no carcinoma identified in the thyroid gland. This diagnosis required synthesis of clinical, biochemical, and histopathologic data. In addition to reviewing prior examples of ectopic MTC, we emphasize the importance of a thorough differential when evaluating calcitonin-secreting tumors in the neck.

## Data Availability

All relevant data are included within the manuscript.
